# Mammary gland tumors in a male Cocker Spaniel

**DOI:** 10.1186/s13028-017-0290-3

**Published:** 2017-04-11

**Authors:** Soon-Chan Kwon, Dae-Young Yoo, Minho Ko, Kwon-Young Lee, Ho-Hyun Kwak, In-Chul Park, In-Koo Hwang, Jung-Hoon Choi, Jin-Young Chung

**Affiliations:** 1 Department of Veterinary Internal Medicine and Institute of Veterinary Science, College of Veterinary Medicine, Kangwon National University, Chuncheon, 200-701 Republic of Korea; 2 Department of Anatomy and Cell Biology, College of Veterinary Medicine, and Research Institute for Veterinary Science, Seoul National University, Seoul, 151-742 Republic of Korea; 3 Department of Anatomy, College of Veterinary Medicine, Kangwon National University, Chuncheon, 200-701 Republic of Korea; 4 Department of Veterinary Surgery, College of Veterinary Medicine, Kangwon National University, Chuncheon, 200-701 Republic of Korea; 5 Department of Veterinary Radiology, College of Veterinary Medicine, Kangwon National University, Chuncheon, 200-701 Republic of Korea

**Keywords:** Adenocarcinoma, Dog, Estrogen receptor, Male, Mammary gland tumor, Progesterone receptor

## Abstract

**Background:**

Mammary gland tumors are the most common tumors in sexually intact female dogs; however, they are rare in male dogs. This study was conducted to investigate the relationship between sexual hormones and mammary gland tumors in a male dog.

**Case presentation:**

A 13-year-old, intact male Cocker Spaniel presented to the Veterinary Teaching Hospital of Kangwon National University, Republic of Korea, with an acute right ruptured caudal abdominal mass. Physical examination revealed a 14 × 14 cm ruptured mass in the right caudal abdomen, as well as a 1.5 × 1.5 cm mass in the first right mammary gland. The estrogen and progesterone concentrations in serum were within normal levels. Total mastectomy was done on the right side mammary glands. Following surgery, the site was fully recovered; however, a mass that had grown to 2 × 2 cm was found in the left fifth mammary gland and a testis tumor was also found over the period of 4 months. Mastectomy was performed on the left caudal mammary gland and castration was also performed. After the final surgery, the dog fully recovered. Histopathological examination of all three masses revealed high grade mammary adenocarcinoma in the mammary gland and the testis was diagnosed as Leydig cell adenoma. Immunohistochemical analysis revealed that the estrogen and progesterone receptors were expressed on limited cells in mammary and testis tumors.

**Conclusion:**

The results of this study suggest that mammary tumors and testes tumors can occur in male dogs without relationship to female sexual hormone.

## Background

Mammary gland tumors are the most common tumors in sexually intact female dogs [1]. Most mammary gland tumors in female dogs are of epithelial origin, and approximately 50% are malignant [2, 3]. The main suspected risk factors for mammary tumors are age, hormone exposure, and breed. Mammary tumors affect middle-age and older dogs, and tend to be more common in smaller breeds [1]. Previous studies suggested that early spayed female dogs have reduced risk of mammary gland tumor development, and that those spayed prior to the first estrus have only a 0.5% risk of mammary gland tumors as compared to intact females. However, there are no significant benefits of ovariohysterectomy after 4 years of age [4]. These results showed that blocking heat cycles in dogs can prevent mammary gland tumors, which suggests that ovarian hormones are important factors influencing the formation mammary gland tumors. Indeed, it has been reported that exposure to exogenous or pharmacologic hormones such as progestin and estrogen increase the risk of developing mammary tumors in dogs [5]. However, not all mammary tumors have reactivity with hormonal receptor antibody, with only approximately 50–77% of epithelial mammary gland tumors expressing hormonal receptors [6]. These findings showed that exposure to factors other than sexual hormones can involve mammary gland tumors in some cases.

Mammary gland tumors are rare in male dogs, as indicated by their being 62 times less likely to develop mammary gland tumors than female dogs [7]. Breast cancer in men is also uncommon in humans, accounting for only 1% of all diagnosed breast cancers [8]. The possible risk factors of breast cancers in men include increasing age, testicular injury, obesity, gynecomastia, Klinefelter’s syndrome and a family history of breast cancer [9, 10]. Accordingly, sexual hormones are a suspected factor of breast cancer in men, and various studies of the relationship between breast cancer in men and sexual hormones have been conducted [11, 12].

The present study was conducted to investigate the relationship between sexual hormone and mammary gland tumors in a male dog, which is especially rare in veterinary medicine.

## Case presentation

A 13-year-old, intact male Cocker Spaniel presented to the Veterinary Teaching Hospital of Kangwon National University, Republic of Korea with an acute right ruptured caudal abdominal mass. Three months before coming to Kangwon National University, a mass was found and developed for three months. Physical examination revealed a 14 × 14 cm ruptured mass in the right caudal abdomen found, as well as a 1.5 × 1.5 cm mass in the first right mammary gland. Hematological analysis revealed moderate neutrophilia. Abnormality in the serum biochemistry included elevated gamma-glutamyl transferase and alkaline phosphatase. The estrogen (42.5 pg/ml: reference range is 31.5–65.4 pg/ml) and progesterone (<0.2 ng/ml: <1.0 ng/ml in the anestrus phase) levels in the serum were within the normal ranges. Assessment of estrogen was performed by chemiluminescent immunoassay in IDEXX Estradiol by RIA, while progesterone was assessed using an IMMULITE kit (IDEXX Reference Laboratories, Westbrook, ME, USA). Nothing remarkable were observed upon thoracic radiography; however, two superficial masses were identified during abdominal radiography. Abdominal ultrasonography demonstrated that there were no abnormal changes in the regional lymph nodes. Total mastectomy of the right side mammary glands was conducted. After surgery, the surgery site was fully recovered; however, a mass was found in the left fifth mammary gland that had grown to 2 × 2 cm over 119 days, as well as a testis tumor. The mass was removed and castration was performed. The dog fully recovered and there was no recurrence on the resident left mammary region as of 750 days after the last surgery.

**Fig. 1:**
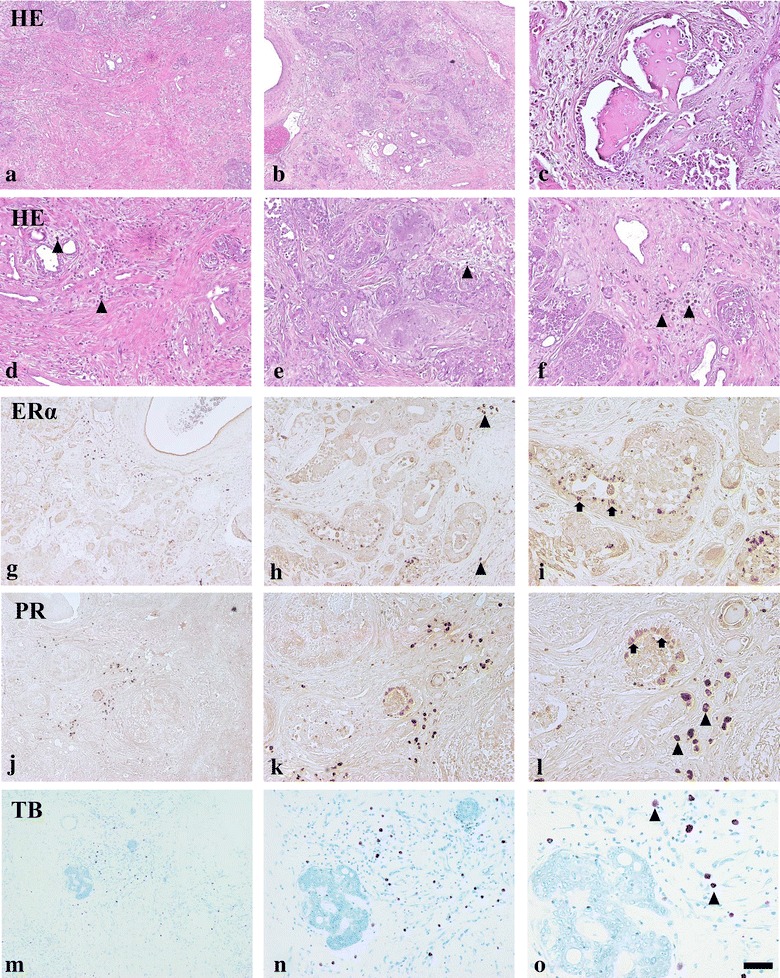
Low and high magnification photos of various regions of the mammary tumor, HE. Desmoplasia (**a**, **d**), chondroid components (**b**, **e**), osseous tissues adjacent duts (**c**) and diffused mast cells (*arrowheads*) in stroma (**f**). Immunohistochemistry of ERα (**g**–**i**) and PR (**j**–**l**) in the tumor. *Toluidine blue* (TB) staining for mast cells (**n**–**o**). *Arrows* indicate epithelial cells. *Scale bars* 250 µm (**a**, **b**, **g**, **j**, **m**), 100 µm (**c**–**f**, **h**, **k**, **n**) and 50 µm (**i**, **l**, **p**)

Microscopic examination of these three mammary gland masses revealed a moderate to high grade invasive mammary carcinoma in a benign mixed tumor [3, 13, 14]. The masses were uncapsulated and exhibited stromal invasion in some locations (Fig. 1a–c). Tumor cells were pleomorphic, ranging from columnar to cuboidal to more polygonal, forming cords, variably-sized nests or glands (Fig. 1e). These cells had scant to moderate cytoplasm with moderate anisokaryosis and coarse to hyperchromatic nuclei. Variable amounts of chondroid and osseous matrices and diffused mast cells were observed within the supporting stromal connective tissue of the mass (Fig. 1d–f).

**Fig. 2:**
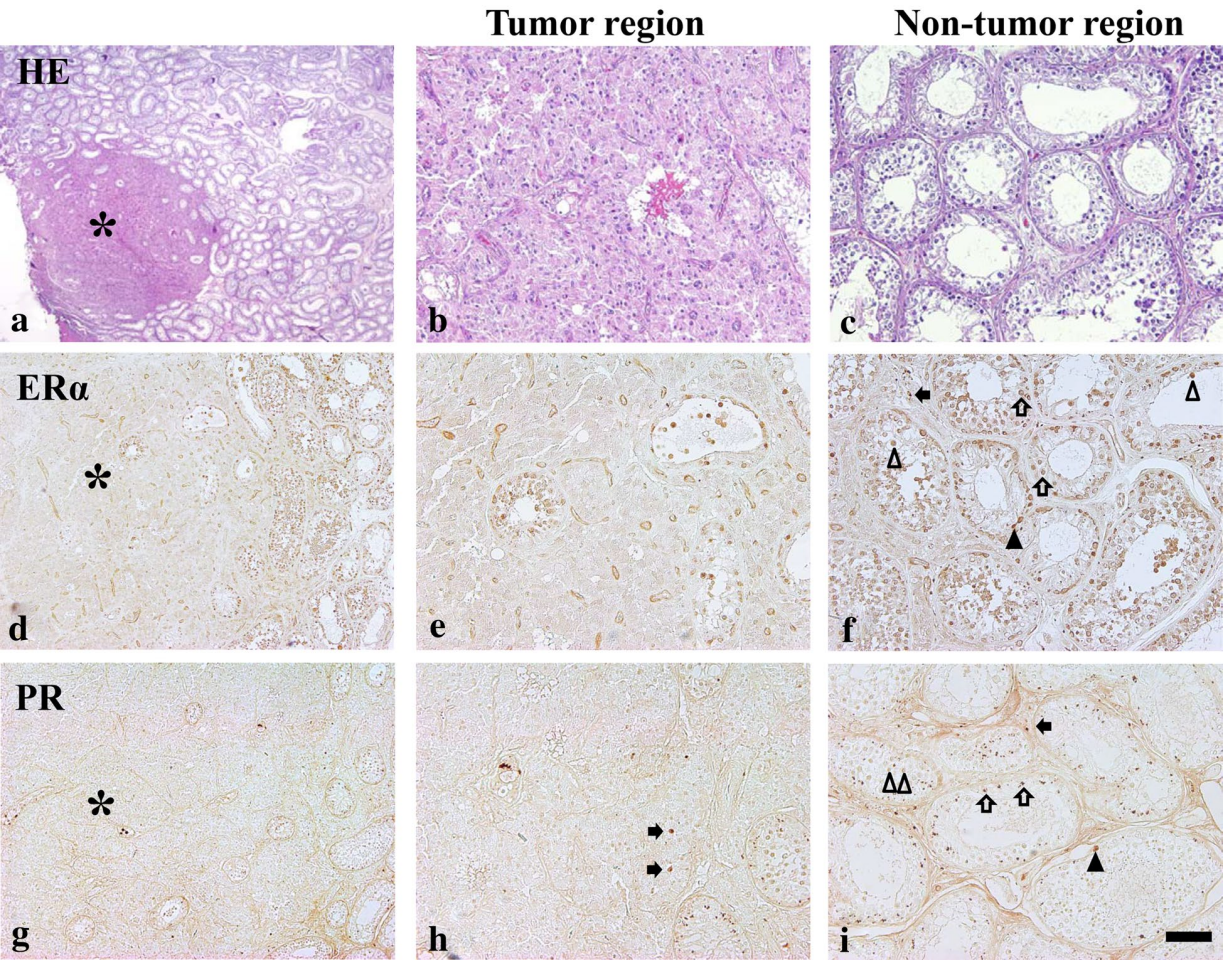
Leydig cells adenoma in the testis (**a**), HE. High magnification photos of tumor region (*asterisk*, **b**) and non-tumor region (**c**). Immunohistochemistry of ERα (**d**, **e**) and PR (**g**–**i**) in the tumor and non-tumor regions of the testis. *Arrowheads* (spermatogonium), *arrows* (Leydig cells), *open arrowheads* (spermatocytes), *open arrows* (spermatids). *Scale bars* 400 µm (**a**), 200 µm (**d**, **g**), 100 µm (**b**, **c**, **e**, **f**, **h**, **i**)

The microscopic features of the masses in the testis were revealed as Leydig cells adenoma and showed characteristic trabecular structures separated by thin fibrovascular structures. These neoplastic cells had an epithelial or columnar amphophilic cytoplasm with monomorphic round nuclei. Degenerating seminiferous tubules were also observed in the tumor region. However, mitotic features were not seen in tumor regions (Fig. 2a, b). No significant morphological changes in seminiferous tubules or interstitial tissues were observed in non-tumor regions (Fig. 2c).

For immunohistochemical analysis, estrogen receptor alpha (ERα, 1:200, anti-estrogen receptor alpha antibody [E115], Abcam, Korea) and progesterone receptor (PR, 1:200, anti-progesterone receptor antibody [PR-AT 4.14], Abcam, Korea) were used in three mammary tumors and testis.

In the mammary tumor, ERα-immunoreactivity was detected in the tumor tissues; however, only a few epithelial cells adjacent to the duct and invasive region were immunopositive for ERα (Fig. 1g–i). In the stromal tissue of the mass, some darkly stained large ERα-immunoreactive cells were observed (Fig. 1g–i). These cells were identified as mast cells based on their morphology and the results of toluidine blue staining (Fig. 1m–o). PR- immunoreactivity was also observed in mammary tumor tissues with similar patterns as for ERα (Fig. 1j–l).

ERα-immunoreactivity was detected in the spermatogonia, spermatocytes, spermatids and Sertoli cells of the seminiferous tubule in both tumor and non-tumor regions. Some ERα-immunoreactive-Leydig cells were also observed in the non-tumor regions. However, ERα-immunoreactive Leydig cells were not observed in the tumor region (Fig. 2d, e). PR-immunoreactivity was also observed in the seminiferous tubules of tumor and non-tumor regions (Fig. 2g–i). Spermatocytes and some Leydig cells were darkly stained with PR, and round spermatids were weakly stained with PR in the non-tumor region (Fig. 2h, i). Scant PR-immunoreactive spermatogonia were also observed (Fig. 2i). Most Leydig cells in the tumor region were not positive for PR antibody, except for a few PR-immunoreactive Leydig cells in the boundary of tumor and non-tumor regions **(**Fig. 2h).

## Conclusions

The influence of sexual hormones in tumorigenesis has been investigated in various species, including dogs [1]. Sexual hormones, including estrogen and progesterone, are essential for physiological mammary growth and development, not only during pregnancy, but throughout the reproductive cycle [15]. The effects of these hormones are mediated via binding to their own receptors [16]. It can be assumed that changes in both ER and PR in any tissue indicate that there are estrogen and progesterone hormonal effects on related tissues [17]. Accordingly, many studies have investigated the expression of ER and PR in mammary gland tumors [16, 17], and hormonal therapies using hormone receptor antagonists have been attempted in mammary gland tumors based on these studies. Surgical excision is the first line treatment of veterinary mammary tumors. However approximately one-third of these tumors will recur and metastasize [18]. Therefore to prevent recurrence and metastasis, other trials, including chemotherapy, should be considered. One study showed that PR antagonist had PR expression related inhibiting effects on proliferation of canine mammary carcinoma cells [19].

Contrary to these findings, some studies have also shown that estrogen levels in inflammatory mammary carcinoma in female dogs were lower than when compared to animals with other carcinoma subtypes [20]. These results conflict with the opinion that sexual hormones influence tumorigenesis in mammary gland tumors. Therefore it is doubtful that early spaying in dogs can decrease the risk of mammary tumors. Actually, the strength in evidence for a preventive effect of neutering has been questioned [21]. Some groups have shown that ERα is down-regulated in high grade breast cancer. The expression of ERα and PR is related to the clinical and biological specificity of the breast cancer [22, 23]. Based on these results, we can assume that the grade of the mammary gland tumors is an important factor in expression of ERα and PR in mammary gland tumors.

Breast cancer in men is rare, and most instances have been diagnosed as malignancies [9]. Breast cancer in men has also been considered to be related to estrogen and progesterone, with approximately 65–80 and 90% of breast cancer cases in men being ER and PR positive, respectively [24, 25]. However, under similar conditions, we can assume that 20–35% of breast cancer in men is not associated with ER and that 10% of breast cancer in men is not associated with PR.

Few studies have investigated mammary gland tumors in male dogs, and only one has reported their diagnosis in male dogs with a subsequent assessment of these dogs [7]. In this study, seven of eight dogs had benign tumors, while only one dog suffered from malignant tumor. There were also no recurrences in the seven dogs with benign tumors, but one dog with malignant tumor developed local recurrence. Identification of the new tumor was noted approximately 115 days after the original diagnosis in this dog. ER expression was intense and occurred in neoplastic cells, while PR expression was less intense in neoplastic cells in most dogs. Unfortunately in this study, they could not confirm the relationship between castration and mammary tumors in male dogs [7].

In the present study, a male dog presented with mammary tumors. Following the first mastectomy, high grade mammary adenocarcinoma was diagnosed. However, a new mammary gland tumor occurred after 119 days as well as a testis tumor. Upon histopathological examination, the testicular tumor was diagnosed as Leydig cell adenoma. There had been no recurrence as of 750 days after surgical removal. However, these findings are not sufficient to explain the relationship between castration and mammary tumors in this case or in male dogs. In this study, there was no clear expression of ER and PR in neoplastic cells of mammary tumors and testis tumors. These results conflict with previous reports that estrogen is highly produced in the Leydig cell adenoma in some cases [26]. We can only assume that the high grade adenocarcinoma in mammary glands affected the expression of ER and PR in neoplastic cells.

This study confirmed the presence of malignant mammary tumors and Leydig cell adenoma of the testis in one male dog. The results suggests that mammary tumors and testes tumors in male dogs can occur without relationship to female sexual hormones.

## Abbreviations

ER: estrogen receptor
ERα: estrogen receptor alpha
PR: progesterone receptor
